# Effect of Chitosan-Based Biodegradable Nasal Packs on Eustachian Dysfunction after Septoplasty

**DOI:** 10.1055/s-0045-1809433

**Published:** 2025-09-10

**Authors:** Halil Elden, Mahmut S. Yilmaz, Ahmet Cihan, Ebru M. Guven, Ahmet Kara

**Affiliations:** 1 Department of Otorhinolaryngology, Sakarya University Training and Research Hospital, Sakarya, Türkiye; 2 Department of Anatomy, Sakarya University Faculty of Medicine, Sakarya, Türkiye

**Keywords:** middle ear effusion, nasal surgery, acoustic impedance tests, auditory tube, splints

## Abstract

**Introduction:**

Bioabsorbable packs, which are frequently used after nasal surgery, have been recently deemed more comfortable than removable packs. If these packs cause lower levels of eustachian dysfunction than silicone nasal splint with integral airway, they would be a good alternative for nasal packing after septoplasty.

**Objective:**

To compare the effects of chitosan-based biodegradable nasal packs and silicone nasal septal splints with integral airway on the ventilation and pressure of the middle ear after septoplasty in patients with normal otoscopic examination.

**Methods:**

Patients who underwent septoplasty for nasal septal deviation with otoscopically-normal tympanic membranes and bilateral, normal type-A preoperative tympanograms were included. Following surgery, the patients were randomized into two groups (group P: chitosan-based biodegradable nasal packs; group S: silicone nasal septal splint).

**Results:**

The tympanometric pressures of all patients in both groups decreased, but more so in group S in the first 24 hours following surgery. These pressure differences between the 2 groups in the first 48 hours following surgery were statistically significant.

**Conclusion:**

The present study demonstrated that chitosan-based biodegradable nasal packs would be a good alternative for nasal packing after septoplasty.

## Introduction

Septoplasty is one of the surgeries most frequently performed for the treatment of nasal obstruction in otorhinolaryngology clinics. Following surgery, temporary eustachian tube (ET) dysfunction is a common problem due to nasal packing, and it is believed to be caused by the absence of nasal airflow and inflammatory mediator release. 1 2 This problem is more common when using packs that completely block the nasal passage. Studies 3 have shown that commonly used silicone nasal septal splints with integral airways cause lower levels of nasal obstruction and negative pressure in the middle ear because they enable nasal airflow through an integral airway and provide septal support.

Bioabsorbable packs, which are frequently used after nasal surgery nowadays, are thought to be more comfortable than removable packings. One of the absorbable packs is PosiSep X (Hemostasis, LLC, St. Paul, MN, United States) which is chitosan-based and non-synthetic. It is a biologically inert and self-dissolved material that enhances wound healing and minimizes bleeding. 4 Recently, it began to be used after endoscopic sinus surgery and septoplasty in our clinic. Although it is used frequently, up to date, there is no study in the literature on its effect on middle ear ventilation and pressure following nasal surgery. Therefore, we aimed to compare the effects of PosiSep X and silicone nasal septal splints with integral airway on the ventilation and pressure of the middle ear after septoplasty in patients with normal otoscopic examination.

## Methods

The present study was conducted after the approval of the institutional Ethics Committee (approval date: July 5th, 2021; no.: 115). Signed informed consent was obtained from all participants. Patients who underwent septoplasty for nasal septal deviation with otoscopically-normal tympanic membranes and bilateral, normal type-A preoperative tympanograms were included. Patients aged under 18 years and those who had turbinate or paranasal sinus pathologies and allergic rhinitis were excluded. Following surgery, the patients were randomized into two groups, one comprised of patients who were submitted to the application of silicone nasal septal splint with integral airway (Unosplint, Genco Tibbi Cihazlar San. Tic. Ltd., Konak, İzmir, Turkey) (group S) the other group comprised of patients who were submitted to the application of hemostat dressing/intranasal splint (PosiSep X, Hemostasis, LLC) (group P) for the nasal packing. The Unosplint was completely removed on the second postoperative day, as we have been doing for a long time in our clinic, while, for PosiSep X, only the portions around the nasal nares were aspirated for cleaning, without removing the entire pack. The middle ear pressure (MEP) of the included patients was measured pre- and postoperatively through tympanometry. Alterations in MEP were also measured pre- and postoperatively through tympanometry using an impedance audiometer (AZ-26, Interacoustics A/S, Middelfart, Denmark). Tympanometry was repeated 24 hours after surgery, just before the removal of the nasal packs on the second day, and, finally, on the seventh postoperative day. The results for each ear were analyzed separately. The tympanograms were classified as originally described by Jerger. 5

Statistical analyses were performed using the IBM SPSS Statistics for Windows (IBM Corp., Armonk, NY, United States) software, version 22.0. The Chi-squared (χ ^2^ ) test was used to compare the categorical variables. The MEP level variables were not normally distributed according to the Kolmogorov–Smirnov normality test. Therefore, nonparametric tests were used to compare the MEP values. The Mann-Whitney U test for two independent samples was used to compare the continuous variables regarding groups P and S. The Friedman test and the Wilcoxon signed-rank test with a Bonferroni correction were used to compare the tympanometric pressure preoperatively and on the first, second, and seventh postoperative days pressures. Repeated measures two-way analysis of variance (ANOVA) was used to compare the alterations in pressure between the two groups. The continuous variables were expressed as median and interquartile range (IQR) values. Values of *p* value < 0.05 were considered statistically significant.

## Results

The present study included 60 (28 male and 32 female) patients, with 30 participants in each group. There were no statistically significant differences between the two groups in terms of age and gender ( Table 1 ).

**Table 1:** Demographic analysis of the study sample

		PosiSep X group	Silicone nasal septal splint group	*p* -value
Mean age in years		31.53 ± 10.29	32.66 ± 8.90	0.33
Gender: n (%)	Female; male	15 (50) 15 (50)	13 (43.3) 17 (56.7)	0.605
Tobacco use: n (%)	Negative; positive	25 (83.4) 5 (16.7)	21 (70) 9 (30)	0.222

The tympanometric pressures decreased in all patients in both groups, but more so in group S in the first 24 hours following surgery. After 24 hours, the MEP started to increase in both groups, and it almost reached the preoperative values by the 48th hour in group P. These pressure differences between the 2 groups in the first 48 hours following surgery were statistically significant. In both groups, the MEP returned to the preoperative values by the seventh postoperative day, except for one patient in group S.

**Fig. 1:**
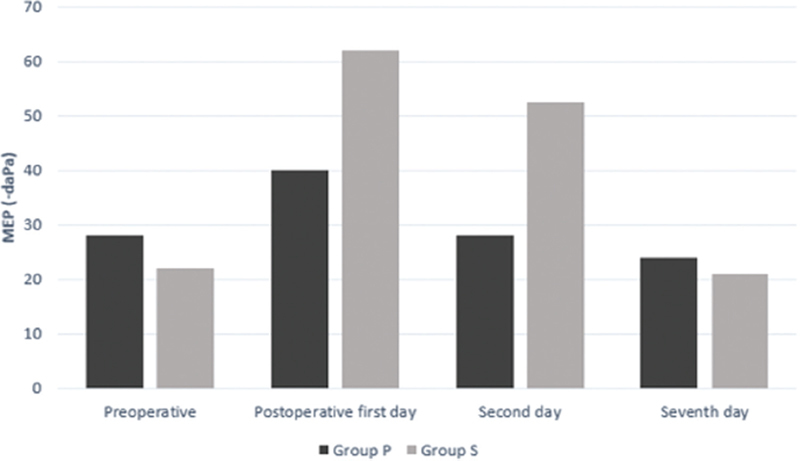
|Graph showing the changes in middle ear pressure in the right ears of groups P (patients with PosiSep X [Hemostasis, LLC]) and S (patients with silicone nasal septal splints).

All MEP values ≤ -100 daPa (by definition, classified as type-C tympanograms) were considered pathological. Pathological decreases in the MEP of at least one ear were observed in 5 patients in group P and in 2 patients in group S 48 hours after surgery, but this difference was not statistically significant ( Figs. 1 2 ; Table 2 ).

**Table 2:** Tympanometric pressures measured preoperatively and on the first, second, and seventh postoperative days

Tympanometric pressure		PosiSep X group	Silicone nasal septal splint group	*p* -value ^a^
Right ear	Preoperative; postoperative: first day; second day; seventh day; *p* -value; ^b^ *p* -value; ^c^	-28.0 [13]; -40.0 [37]; -28.0 [30]; -24.0 [13]; 0.017; < 0.001	-22.0 [16]; -62.0 [113]; -52.5 [64]; -21.0 [16]; < 0.001	0.103; 0.021; 0.001; 0.466
Left ear	Preoperative; postoperative: first day; second day; seventh day; *p* -value; ^b^ *p* -value; ^c^	-24.0 [20]; -36.0 [32]; -32.0 [28]; -26.0 [21]; 0.001; 0.014	-22.0 [19]; -56.5 [93]; -45.0 [58]; -22.0 [19]; < 0.001	0.529; 0.006; 0.219; 0.468

**Notes:**^a^ Results of the comparison between the two groups. ^b^ Results of the comparison of the pressures measured preoperatively and on the first, second, and seventh postoperative days. ^c^ Results of the comparison between the two groups regarding alterations in pressure.

**Fig. 2:**
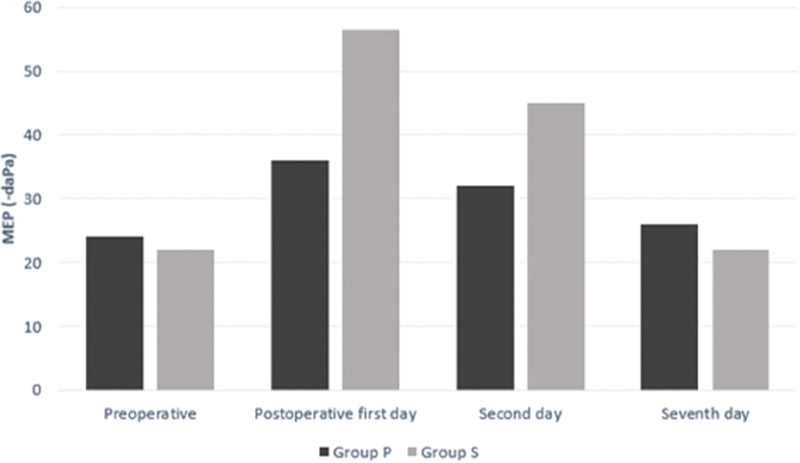
Graph showing the changes in middle ear pressure in the left ears of groups P and S.

In group P, 48 hours postoperatively, 2 patients presented bilateral type-C tympanograms, and 3, unilateral type-C tympanograms, but type-B tympanograms were not observed in any of the patients. In group S, 1 patient presented a unilateral type-C tympanogram, and another patient, a bilateral type-C tympanogram 48 hours following surgery. Likewise, no type-B tympanograms were observed in any of the patients in group S.

Tympanometry performed 5 days after the removal of the nasal packs revealed normal type-A tympanograms for all ears in group S. Although 29 patients returned to normal type-A tympanograms in group P, 1 patient still presented a type-C tympanogram in both ears. No patient in either group presented a type-B tympanogram during the study period. Furthermore, no otoscopic or tympanometric evidence of middle ear effusion was observed in any patient in either group, including those with type-C tympanograms ( Table 3 ).

**Table 3:** Tympanogram types of the two groups measured on the first, second, and seventh days postoperatively

		PosiSep X group (n)			Silicone nasal septal splint group (n)			
Tympanogram type		A	B	C	A	B	C	*p* -value*
Right ear	First postoperative fday	24	0	6	27	0	3	0.278
	Second postoperative day	25	0	5	28	0	2	0.228
	Seventh postoperative day	29	0	1	30	0	0	0.313
Left ear	First postoperative day	24	0	6	26	0	4	0.488
	Second postoperative day	28	0	2	29	0	1	0.554
	Seventh postoperative day	29	0	1	30	0	0	0.313

**Note:** *Pearson's Chi-squared test.

## Discussion

The ET is a functional passage between the middle ear and the nasopharynx that provides ventilation to the middle ear. It is frequently involved in pathological processes affecting the nasal, paranasal and nasopharyngeal cavities. 6 Salvinelli et al. 7 reported that chronic nasal obstruction is a frequent cause of ET dysfunction which can lead to middle ear hypoventilation. The inflammatory reaction and edema in the nasopharyngeal mucosa due to surgical trauma and packing is believed to cause ET dysfunction.

Nasal packs are used to prevent bleeding and support the septal mucoperichondrial flap to minimize the risk of septal hematoma and synechiae following nasal surgery and epistaxis. Several packing materials, including vaseline gauze, Merocel (Medtronic plc, Minneapolis, MN, United States), and septal splints, have been used following septoplasty, as they all present their own advantages. It is expected that the insertion and removal of the nasal packing does not cause pain. Furthermore, it should be easily removable and cause less nasal fullness.

Negative MEP measured by tympanometry is a sign of ET dysfunction, a complication caused by nasal packings after septoplasty that is usually reversible. However, it causes patient discomfort and dissatisfaction following surgery. In a study using bilateral anterior nasal gauze packs following septoplasty, Thompson and Crowther 8 found 46% of patients with a negative MEP, lower than -50 dPa. Moreover, Mc Curdy 1 reported a negative pressure ≤ -100 daPa in 25% of 99 ears 3 days after applying bilateral anterior nasal packing. 1 In the current study, a type-C tympanogram was obtained in at least 1 ear in 5 patients in group P and in 2 patients in group S 48 hours after septoplasty.

Merocel and silicone splint have recently become the preferred material among packing alternatives following septoplasty. Since it enables nasal breathing through an integral airway, the silicone nasal septal splint with integral airway is believed to cause lower levels of negative pressure in the middle ear and increase patient comfort postoperatively. Several studies 3 9 9 10 comparing Merocel and silicone splints after septoplasty have shown that silicone splints with integral airway cause lower levels of ET dysfunction and aural fullness than Merocel postoperatively.

Due to patients' stress and fear of packing removal, bioabsorbable materials that do not require removal have been recently used as packings after nasal surgery. Furthermore, absorbable packs are more comfortable than removable packs. 11 Among them, the preferred packs are Nasopore (Stryker Corporation, Kalamazoo, MI, United States), a synthetic polyurethane pack, and PosiSep X, a chitosan-based non-synthetic nasal pack. Both are self-dissolved and biologically inert materials. They also enhance wound healing and minimize bleeding. Previous studies 11 12 have shown that absorbable packs, which reduce pain and discomfort during packing and removal following nasal surgery, are efficient and safe. Khafagy and Maarouf 13 compared the clinical outcomes of the use of Nasopore and PosiSep X after functional endoscopic sinus surgery. They reported that both packs are efficient and safe regarding mucosal healing and bleeding control. PosiSep X showed a higher advantage in the first two weeks regarding the amount of the retained material crusting as well as bleeding in their study. 13

Although there are studies on the effectiveness of absorbable packs after nasal surgery, there is no study in the literature on their effects on ET function following nasal surgery. Our results show that PosiSep X causes lower levels of decrease in MEP in the first 48 hours following surgery compared to silicone splint. Although it decreased in the first 24 hours, we observed that the MEP in group P tended to increase to normal values immediately afterwards. But our results show that the return to normal MEP values in the splint group took up to 1 week following surgery. We think that this is because PosiSep X causes lower levels of pressure in the nasal cavity, although a silicone nasal splint with an integral airway enables nasal breathing.

## Conclusion

Temporary aural fullness and ET dysfunction, which lead to patient discomfort and dissatisfaction due to nasal packing after septoplasty, are a common problem. The current study demonstrated that PosiSep X caused lower levels of ET dysfunction than silicone nasal splint with integral airway. Moreover, PosiSep X does not require removal. Therefore, we think it would be a good alternative for nasal packing after septoplasty.

## References

[1] McCurdy J A Jr Effects of nasal packing on eustachian tube function Arch Otolaryngol 1977 103 09 521 523 901277 10.1001/archotol.1977.00780260051004

[2] Tos M Development of mucous glands in the human Eustachian tube Acta Otolaryngol 1970 70 05 340 350 5505128 10.3109/00016487009181895

[3] Yilmaz M S Guven M Buyukarslan D G Kaymaz R Erkorkmaz U Do silicone nasal septal splints with integral airway reduce postoperative eustachian tube dysfunction? Otolaryngol Head Neck Surg 2012 146 01 141 145 21900536 10.1177/0194599811421595

[4] Hsu K Ericksen M Catalano P Effect of a chitosan-based biodegradable middle meatal dressing after endoscopic sinus surgery: a prospective randomized comparative study Sinusitis 2016 1 3 12

[5] Jerger J Clinical experience with impedance audiometry Arch Otolaryngol 1970 92 04 311 324 5455571 10.1001/archotol.1970.04310040005002

[6] Bonding P Tos M Middle ear pressure during brief pathological conditions of the nose and throat Acta Otolaryngol 1981 92(1-2):63 69 7315256 10.3109/00016488109133238

[7] Salvinelli F Casale M Greco F D'Ascanio L Petitti T Di Peco V Nasal surgery and eustachian tube function: effects on middle ear ventilation Clin Otolaryngol 2005 30 05 409 413 16232243 10.1111/j.1365-2273.2005.01052.x

[8] Thompson A C Crowther J A Effect of nasal packing on eustachian tube function J Laryngol Otol 1991 105 07 539 540 1875135 10.1017/s0022215100116548

[9] Topal K Kars A Bingol F Effect of intranasal merocel packs, silicone splint, and trans-septal suture after septoplasty on Eustachian dysfunction B-ENT 2020 16 193 196

[10] Şereflican M Yurttaş V Oral M Yılmaz B Dağlı M Is middle ear pressure effected by nasal packings after septoplasty? J Int Adv Otol 2015 11 01 63 65 26223721 10.5152/iao.2015.1085

[11] Yilmaz M S Guven M Elicora S S Kaymaz R An evaluation of biodegradable synthetic polyurethane foam in patients following septoplasty: a prospective randomized trial Otolaryngol Head Neck Surg 2013 148 01 140 144 23112275 10.1177/0194599812465587

[12] Wang J Cai C Wang S Merocel versus Nasopore for nasal packing: a meta-analysis of randomized controlled trials PLoS One 2014 9 04 e93959 24710428 10.1371/journal.pone.0093959 PMC3977961

[13] Khafagy A G Maarouf A M Polyurethane versus chitosan-based polymers nasal packs after functional endoscopic sinus surgery: a prospective randomized double-blinded study Am J Rhinol Allergy 2021 35 05 624 630 33430613 10.1177/1945892420983645

